# Enhancing the antitumor activity of an engineered TRAIL-coated oncolytic adenovirus for treating acute myeloid leukemia

**DOI:** 10.1038/s41392-020-0135-9

**Published:** 2020-04-24

**Authors:** Zixuan Wang, Wenmo Liu, Lizheng Wang, Peng Gao, Zhe Li, Jiaxin Wu, Haihong Zhang, Hui Wu, Wei Kong, Bin Yu, Xianghui Yu

**Affiliations:** 1 National Engineering Laboratory for AIDS Vaccine, School of Life Sciences, Jilin University, Changchun, 130012 China; 2 Department of Hematology, Jilin Province People’s Hospital, Changchun, 130021 China; 3 Key Laboratory for Molecular Enzymology and Engineering, The Ministry of Education, School of Life Sciences, Jilin University, Changchun, 130012 China

## Abstract

The use of oncolytic viruses has emerged as a promising therapeutic approach due to the features of these viruses, which selectively replicate and destroy tumor cells while sparing normal cells. Although numerous oncolytic viruses have been developed for testing in solid tumors, only a few have been reported to target acute myeloid leukemia (AML) and overall patient survival has remained low. We previously developed the oncolytic adenovirus rAd5pz-zTRAIL-RFP-SΔ24E1a (A4), which carries the viral capsid protein IX linked to tumor necrosis factor-related apoptosis-inducing ligand (TRAIL) and results in increased infection of cancer cells and improved tumor targeting. To further improve the therapeutic potential of A4 by enhancing the engagement of virus and leukemia cells, we generated a new version of A4, zA4, by coating A4 with additional soluble TRAIL that is fused with a leucine zipper-like dimerization domain (zipper). ZA4 resulted in enhanced infectivity and significant inhibition of the proliferation of AML cells from cell lines and primary patient samples that expressed moderate levels of TRAIL-related receptors. ZA4 also elicited enhanced anti-AML activity in vivo compared with A4 and an unmodified oncolytic adenoviral vector. In addition, we found that the ginsenoside Rh2 upregulated the expression of TRAIL receptors and consequently enhanced the antitumor activity of zA4. Our results indicate that the oncolytic virus zA4 might be a promising new agent for treating hematopoietic malignancies such as AML.

## Introduction

Acute myeloid leukemia (AML) is a myeloid hematopoietic stem/progenitor cell malignant disease that is characterized by the clonal expansion of primitive cells with abnormal differentiation.^1^ Although a number of patients achieve complete remission after first-line induction and consolidation chemotherapy, the majority of them experience relapse.^2–4^ In addition, ~30–40% of AML patients are refractory to the initial therapy. Thus, more effective therapies are urgently needed to improve the outcomes of AML patients. Oncolytic viruses have recently emerged as a promising strategy for the treatment of various tumors, because they replicate only in infected cancer cells but not in normal tissues and are able to infect adjacent cancer cells after selective virus propagation, consequently leading to virus-mediated tumor cell lysis.^5^ Several oncolytic viruses, such as the measles virus,^6^ reovirus,^7^ vesicular stomatitis virus (VSV),^8^ and myxoma virus,^9^ have been used to treat hematologic malignancies in preclinical and clinical studies. Due to their lytic replication and high efficiency of gene transfer, oncolytic adenoviruses have been widely tested in cancer therapy.^10,11^ However, they are rarely used in leukemia treatment, as intravenous (i.v.) injection of an adenovirus type 5 (Ad5)-based oncolytic adenovirus resulted in liver tropism, thus compromising any potential efficacy.^12^ Moreover, leukemia cells express low levels of Coxsackie-adenovirus receptor (CAR), which is an Ad5 receptor, resulting in a low level of Ad5 infection.^13^ Nevertheless, oncolytic adenoviruses expressing therapeutic genes showed enhanced antitumor activity in CAR-expressing B-lymphoblastic leukemia cells.^14^

Previously, we designed and constructed a novel oncolytic Ad5 strain (rAd5pz-zTRAIL-RFP-SΔ24E1a; A4) expressing tumor necrosis factor-related apoptosis-inducing ligand (TRAIL), which is coupled to capsid protein IX (pIX) by a synthetic leucine zipper-like dimerization domain (zipper). Thus, A4 carries TRAIL on its surface and is able to target tumor cells.^15^ TRAIL induces apoptosis by binding the death receptors (DR4 and DR5) that are highly expressed on the surfaces of tumor cells.^16,17^ A4 showed significant tumor-targeting capability, reduced liver tropism, and potent antitumor activity.^15^ However, we also found that the amount of TRAIL coupled with the capsid protein on the viral particle surface was less than expected, indicating that A4 needs to be further improved to ensure better efficacy. Previous studies showed that gene therapy based on either recombinant soluble TRAIL (sTRAIL) or native TRAIL showed selective cytotoxicity toward cancer cells. Therefore, we further modified A4 by coating it with a purified TRAIL fusion protein expressed in bacteria (herein named zA4) to enhance its tumor-targeting ability.

As for any monotherapy, tumor cells may show no response to TRAIL-mediated apoptosis due to intrinsic or acquired resistance.^18^ The identification of sensitizing agents capable of overcoming resistance to TRAIL-induced apoptosis may improve the efficacy of TRAIL-mediated therapy.^19^ Ginsenosides are the major active ingredients of ginseng and are known to have multiple effects on the enhancement of intelligence, immune response, metabolism, and cancer prevention and treatment.^20^ The ginsenoside Rh2 is considered to be a promising antitumor molecule that acts through multiple cellular targets and signal transduction pathways.^21^ Rh2 has been shown to induce the expression of death receptors, including Fas, FasL, DR5, and TRAIL, in the HL-60 AML cell line, leading to the induction of apoptosis and differentiation of cancer cells.^22^ Thus, we hypothesized that Rh2 may have the potential to enhance sensitization to TRAIL-induced apoptosis.

In this study, we generated a new version of A4, zA4, to improve the infectivity and therapeutic efficacy of A4 in the treatment of AML. We also evaluated the therapeutic efficacy of zA4 in combination with the ginsenoside Rh2 in treating AML.

## Materials and methods

### Cell lines and cell culture

The cell lines used in this study were purchased from the American Type Culture Collection (ATCC, Manassas, VA), including the human embryonic kidney cell line HEK-293 (ATCC® CRL-1573™), human breast epithelial cell line ZR-75-30 (ATCC® CRL-1504™), human AML cell line THP-1 (ATCC TIB-202), human lymphoblast line MV4-11 (ATCC® CRL-9591™), and human cutaneous T-lymphocyte line H9 (ATCC® HTB-176™). The HEK-293 cell line was cultured in Dulbecco’s modified Eagle’s medium (Invitrogen, Carlsbad, CA) supplemented with 10% heat-inactivated fetal bovine serum (FBS; Gibco-BRL, Grand Island, NY, USA), 100 U/mL penicillin, and 100 mg/mL streptomycin. MV4-11 cells were cultured in Iscove’s modified Dulbecco’s medium (Invitrogen) supplemented with 10% heat-inactivated FBS. Other cell lines were cultured in Roswell Park Memorial Institute 1640 medium (Invitrogen) supplemented with 10% heat-inactivated FBS, 100 U/mL penicillin, and 100 mg/mL streptomycin. All cell lines were authenticated by short tandem repeat profiling <6 months ago, which is when this project was initiated. All cell lines were passaged for 2 months, to ensure fidelity of the cell line identity. All cells were tested for mycoplasma contamination and all of the cell lines in this study were confirmed to have received treatment for mycoplasma contamination.

### Antibodies and reagents

The following antibodies were used: anti-DR4 (307208 for flow cytometry; BioLegend, San Diego,CA; ab8414 for western blotting; Abcam, Cambridge, UK), anti-DR5 (119906 for flow cytometry; BioLegend; ab8416 for western blotting, Abcam), anti-DcR1 (ab2087; Abcam), anti-DcR2 (ab2019; Abcam), anti-CXADR (05-644; Millipore, Billerica, MA), anti-Ad5 (ab6982; Abcam), anti-caspase-3 (sc-56055; Santa Cruz Biotechnology; Santa Cruz, TX), anti-c-caspase-3 (610322; Becton Dickinson, Franklin Lakes, NJ), anti-PARP (100984-T46; Sino Biological, Beijing, China), anti-FLIP (ab56531; Abcam), anti-tubulin (ab15246; Abcam), anti-CD45 (10086-MM05-F; Sino Biological), and anti-CD33 (12238-MM09; Sino Biological). Rh2 (MB6870) was purchased from Meilunbio (Dalian, China).

### Human blood specimens

Human peripheral blood specimens were collected after obtaining informed consent from all subjects. Primary AML cells were obtained from patients at diagnosis and granulocytes were obtained from healthy donors, which were purified by standard Ficoll-Hypaque density centrifugation.

### Flow cytometry analysis

The expression of decoy receptor 1 (DcR1), decoy receptor 2 (DcR2), death receptor 4 (DR4), death receptor 5 (DR5), CAR, integrin αvβ3, and integrin αvβ5 in cell lines and primary AML cells were determined by flow cytometry using the specific antibodies described above. Briefly, the cells were incubated with specific primary antibodies for 1 h and then incubated with fluorescein isothiocyanate-labeled secondary antibodies for 45 min, after which the cells were analyzed by flow cytometry with an Accuri C6 flow cytometer (Becton Dickinson). To assess apoptosis, cells were infected by three types of adenoviruses at a multiplicity of infection (MOI) = 100, collected at 2, 6, and 8 h after infection, and washed with phosphate buffered saline (PBS). Cells were resuspended in binding buffer, stained with Annexin V (0.6 mg/mL) and propidium iodide (5 mg/mL) for 15 min in the dark at room temperature, and then analyzed by flow cytometry with an Accuri C6 flow cytometer.

### Expression and purification of leucine zipper-fused human sTRAIL (zTN)

A leucine zipper fused with human sTRAIL was cloned into the pET28a vector by homologous recombination. The recombinant plasmid was transformed into BL21 *Escherichia coli* (DE). After 16 h of isopropyl-β-d-thiogalactoside induction at 18 °C, the cells were collected, centrifuged at 6000 × *g* for 30 min and resuspended in 50 mM Tris-HCl (pH 8.0). The cells were then disrupted by ultrasonication for 15 min, after which the cell lysate was centrifuged at 10,000 × *g* for 30 min. The supernatant was filtered through a 0.45 μm filter prior to affinity chromatography. The cell lysate was loaded onto a column containing nickel resin pre-equilibrated with wash buffer, followed by washing with ten bed volumes of wash buffer (150 mM NaCl and 50 mM Tris-HCl, pH 8.0) with increasing concentrations of imidazole (20, 50, 75, 125, 250, and 500 mM). The protein was eluted with wash buffer containing 125 mM imidazole and concentrated to a final concentration of ~1 mg/mL using a 3 kDa ultrafiltration tube (Millipore).

### SDS–PAGE, gradually denaturation SDS–PAGE, and western blotting analyses

SDS–polyacrylamide gel electrophoresis (SDS–PAGE) and gradually denaturation SDS–PAGE (GDS–PAGE) analyses were carried out using 13.5% polyacrylamide gels. Samples were denatured by boiling them in 4× SDS sample buffer (62.5 mM Tris-HCl, 2% SDS, 5% mercaptoethanol, 10% glycerol, and 0.002% bromophenol blue) prior to SDS–PAGE analysis. The GDS–PAGE procedure was similar to the SDS–PAGE procedure, except that the samples were not boiled and the proteins were gradually denatured due to the effect of SDS. Cells were treated with Rh2 at different concentrations for 4 h at 37 °C and then incubated with or without sTRAIL for 16 h at 37 °C. Cells were collected and washed once with prechilled PBS and lysed in RIPA buffer for 20 min at 4 °C, followed by the addition of 4× SDS sample buffer. Samples were then subjected to SDS–PAGE after boiling for 10 min, after which the proteins were transferred onto nitrocellulose membranes by semi-dry transfer (Bio-Rad Laboratories, Hercules, CA). After blocking in 5% nonfat milk for 10 min, the membranes were probed with specific primary antibodies overnight at 4 °C. After incubation with secondary antibodies, the immunoreactions were visualized with Tanon High-sig ECL Western Blotting Substrate using a chemiluminescent imaging system (Tanon 4600; Tanon, Shanghai, China).

### Enzyme-linked immunosorbent assay

Monoclonal antibodies (100 μL/well) against the Ad5 hexon protein were coated onto 96-well plates at 4 °C overnight. After blocking with 5% bovine serum albumin/PBST (0.05% Tween-20 in 20 mM PBS) at 37 °C for 1 h, the plates were incubated with the adenoviruses at 37 °C for 2 h. The samples were then incubated with a rabbit polyclonal antibody (1: 2000; ab2435; Abcam) against TRAIL at 37 °C for 1 h. The plates were incubated with a peroxidase-conjugated affinity-purified goat anti-rabbit secondary antibody (1: 500; Proteintech, Rosemont, IL). After each step, the plates were washed three times with PBST. The color visualization was performed using tetramethylbenzidine. The reaction was stopped with 2 M H_2_SO_4_ and analyzed at two wavelengths (450–630 nm) with an ELX800 Universal Microplate Reader (Bio-Rad Laboratories).

### Generation of recombinant adenovirus with zTN

An oncolytic adenovirus carrying the linker (G_4_S)_3_-zipper (R-R34) attached to the C-terminal end of pIX was produced in HEK-293 cells. The infected cells were collected and lysed by multi-gelation three times. After centrifugation at 400 × *g*, the supernatant was incubated with purified zTN protein for 16 h at 4 °C and then purified using CsCl equilibrium density gradient centrifugation, as well as rAd5-zTRAIL-RFP-SΔ24E1a (A3), rAd5pz-zTRAIL-RFP-SΔ24E1a (A4), and rAd5-RFP-SΔ24E1a (A5). The infection unit (IU) titers were determined by the TCID_50_ (tissue culture infection dose 50) method.^23^

### Analysis of adenovirus infection, binding, and internalization

To analyze adenoviral infection, 2 × 10^5^ cells were plated in each well of a 24-well plate and infected with recombinant adenoviruses at an MOI of 100. The red fluorescence protein (RFP) expressed by the oncolytic adenovirus was detected with a fluorescence microscope after 24 h of infection. The cell-binding assay was performed in a 24-well plate seeded with 2 × 10^5^ cells per well. Cells were cultured in serum-free media at 4 °C for 1 h in the presence of 1000 virus particles per cell. Then, the cells were collected for quantitative PCR (qPCR). To measure the vector internalization, cells were plated in a 24-well plate at 2 × 10^5^ cells per well. Cells were cultured in serum-free media at 4 °C for 1 h in the presence of 1000 virus particles per cell and then incubated at 37 °C for 16 h before qPCR was performed.^24^ The viral and total genomic DNA was isolated using the QIAamp DNA Mini Kit (Qiagen, Hilden, Germany) and quantified with a Nanodrop spectrophotometer (Thermo Fisher Scientific, Waltham, MA). Fifty nanograms of total DNA was subjected to qPCR analysis using SYBR Green and 0.2 μM hexon primers and a standard curve was generated with 10^2^–10^7^ Ad particles with a 7900HT Sequence Detection System (Applied Biosystems, Foster City, CA).

### Cell viability assay

Adenovirus-induced cell death was examined by an MTT (3-(4,5-dimethylthiazol-2-yl)-2,5-diphenyltetrazolium bromide) assay. Cell viability was measured after 24 or 72 h of infection with recombinant adenoviruses at various concentrations (MOI = 0, 10, 25, 50, 75, 100, 150, and 200). To detect the effect of the ginsenoside Rh2, THP-1 cells were incubated with Rh2 for 4 h before infection with adenoviruses. The survival ratio (%) was calculated according to the following formula: (experimental group absorbance − background absorbance)/(control group absorbance − background absorbance) × 100%.

### Xenograft tumor model

All animal work was performed in accordance with the Guidelines for the Welfare of Animals in Experimental Neoplasia. Male BALB/c nude mice (3–4 weeks old) (Vital River Laboratories, Beijing, China) were quarantined for 1 week before tumor implantation. All procedures were performed according to institutional guidelines and conformed to the National Institutes of Health guidelines for the ethical use of animals. THP-1 cells (1 × 10^7^) were injected i.v. or subcutaneously into the right flank. Once the tumors had grown to 100–200 mm^3^, or the percentage of THP-1 in peripheral blood mononuclear cells (PBMCs) was ~15–20%, the animals were randomly assigned to different treatment groups. Each group was treated with either PBS or 2 × 10^8^ IU of vector via i.v. injection for five consecutive days. The tumor size was measured using Vernier calipers every 2 days and the tumor volume (mm^3^) was calculated as (length × width^2^)/2. Blood samples were collected on days 14, 19, 21, 28, 35, and 42 from the i.v. mouse model and the percentage of THP-1 cells among mouse PBMCs was determined by anti-CD33/CD45 using an Accuri C6 flow cytometer. All the data used to generate the tumor growth curve were based on the subtraction of the tumor size or THP-1% determined on the day before the treatment of each mouse. All the tested animals were killed at the end of the experiment. Differences in tumor growth were evaluated for statistical significance.

At the end of treatment, three mice from each group were randomly killed. The liver, kidney, and tumor tissues were fixed with paraformaldehyde and paraffin sections were obtained for hematoxylin and eosin (H&E) staining and immunohistochemical (IHC) assays. After high-pressure antigen retrieval, the tumor tissue sections were incubated with primary goat anti-cleaved caspase-3 or anti-Ad5 antibodies at 20 μg/mL. After incubation with a horseradish peroxidase-conjugated anti-rabbit secondary antibody, the signals were detected with DAB (Sigma-Aldrich, St. Louis, MO) and enhanced with an avidin–biotin reaction ABC kit (Vector Laboratories, Burlingame, CA). The slides were then counterstained with hematoxylin.

Mice with subcutaneous xenografts of THP-1 cells were i.v. injected with 2 × 10^10^ IU of virus/mouse. After 72 h, the mice were subjected to whole-body bioluminescent quantification using FX PRO (Kodak, Rochester, NY) and then killed after observation. The tissue was extracted for the biodistribution analysis of recombinant adenoviruses in each organ. DNA was extracted from tissues using the QIAamp DNA Mini Kit (Qiagen) according to the manufacturer’s instructions and then quantified using a Nanodrop spectrophotometer (Thermo Fisher Scientific). Finally, viral genome-containing DNA was quantified via qPCR using SYBR Green with a 7900HT Sequence Detection System (Applied Biosystems).

### Statistical analysis

The values are expressed as the mean ± SD. Statistical analysis was carried out using one-way analysis of variance or an unpaired *t*-test followed by the Newman–Keuls test. *P*-values < 0.05 were considered significant. The overall survival probability was estimated using the Kaplan–Meier method and the statistical analysis was performed using the log-rank test with GraphPad Prism 8.

## Results

### Assessment of the expression of TRAIL and Ad5 receptors in AML cell lines and primary AML cells

**Fig. 1:**
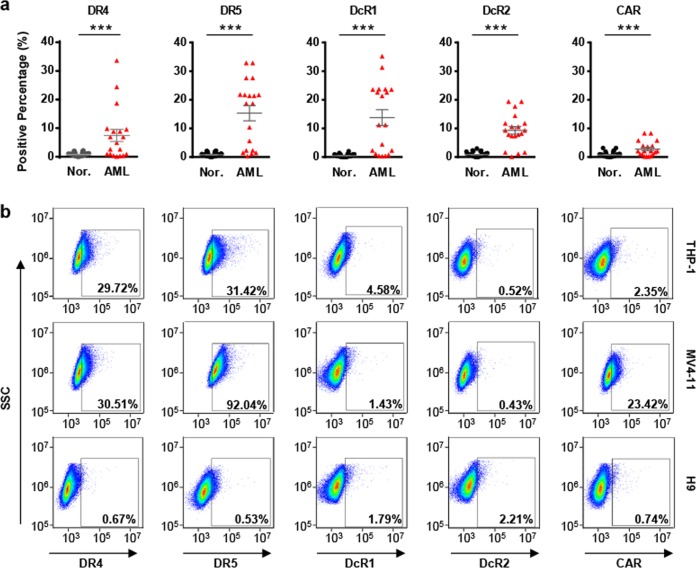
Expression profiling of TRAIL and Ad5 receptors in cells derived from clinical samples and cell lines. **a** Expression levels of DR4, DR5, DcR1, DcR2, and CAR in AML specimens (*n* = 19) and healthy donors (*n* = 68) were determined by flow cytometry. **b** Expression levels of DR4, DR5, DcR1, DcR2, and CAR in THP-1, MV4-11, and H9 cells were determined by flow cytometry. Error bars represent the SEM. *P < 0.05; **P < 0.01; ***P < 0.001

Before analyzing the infection and replication of the oncolytic Ad vectors, we assessed the expression of TRAIL receptors (death receptors, DR4 and DR5; decoy receptors, DcR1 and DcR2) and adenovirus receptors (CAR; integrin αvβ3 and integrin αvβ5) on primary AML cells, which were isolated from AML patient samples and subjected to flow cytometry, to identify the CD45^+^ CD33^+^ primary AML cells. The expression levels of TRAIL receptors varied widely among the patients. As shown in Fig. 1a, over 50% of the 19 AML patients expressed moderate levels of DR4, DR5, DcR1, or DcR2, whereas 4 out of 19 AML patients expressed relatively high levels of CAR (>5% positive cells) and 1 out of 19 patients expressed relatively high levels of integrin αvβ5 (>5% positive cells) (Fig. 1a and Supplementary Table S1). The expression of these receptors was also detectable in the two AML cell lines, MV4-11 and THP-1 (Fig. 1b). Both cell lines expressed relatively high levels of death receptors, but decoy receptors (DcR1 and DcR2) were expressed at low levels. THP-1 and MV4-11 cells expressed low levels of CAR, although 23.42% of MV4-11 cells were positive. In contrast, TRAIL and adenovirus receptors could not be detected in granulocytes from nearly all 68 normal human controls (Supplementary Table S2). H9, a normal human T-lymphocyte cell line that was used as a negative control, showed the lowest expression of TRAIL and adenovirus receptors among the assessed cell lines. Because of the high expression of DR4 and DR5 in primary AML cells, as well as in AML cell lines, we assumed that a gene therapy approach that used TRAIL-coated adenovirus as an oncolytic vector might result in therapeutic efficacy by inducing apoptosis.

### Generation of TRAIL-coated oncolytic adenovirus

**Fig. 2:**
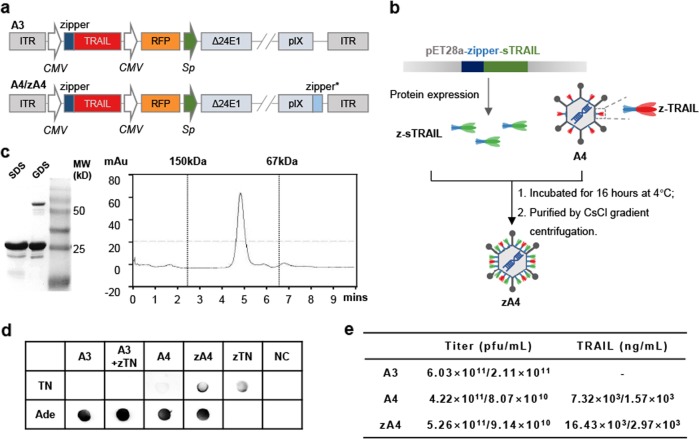
Construction of the recombinant zA4 vector. **a** Schematic diagram of recombinant Ad constructs. **b** Schematic diagram of the strategy used for generating the zA4 virus. **c** Biochemical analysis of the purified zTRAIL using SDS–PAGE and GDS–PAGE followed by Coomassie Blue staining (left) or molecular determination of the purified zTRAIL using HPLC TSK gel G2000 (TOSOH, Tokyo, Japan) (right). Purified IgG (150 kDa) and bovine serum albumin (67 kDa) were used as standards for HPLC analysis. **d** Dot blotting was used to determine the expression of TRAIL on the surface of viral capsids with a polyclonal anti-Ad capsid antibody. **e** TRAIL levels on the surface of the viral capsid were quantified by ELISA. Three independent experiments were conducted to average the values

To generate a targeted oncolytic virus, the adenovirus A3 was engineered to express a TRAIL-zipper and capsid pIX-zipper fusion under the control of two promoters, which was named A4 (Fig. 2a). TRAIL was consequently present on the surface of A4 after being linked to pIX by dimerization of the zipper domains. Although A4 showed good targeting and antitumor activity in TRAIL-sensitive breast cancer cells, we found that only ~17% of pIX was linked to TRAIL.^15^ To enhance AML cell targeting and killing efficacy, we used a new strategy that involved expressing sTRAIL fused to the zipper protein in *E. coli*, which was termed zTN. Then, zTN was incubated with A4 at 4 °C for 16 h. The zTN-coated A4 (named zA4) was purified by density gradient centrifugation (Fig. 2b). Analysis of the purified zTN by SDS–PAGE showed a major band with a molecular mass of ~25 kDa, which was consistent with the predicted molecular weight of zTN. The gradual denaturation SDS–PAGE (GDS–PAGE) showed two protein bands at ~25 kDa and ~75 kDa, indicating that at least a fraction of zTN was present in the trimer isoform on the GDS gel. In line with this, high-performance liquid chromatography (HPLC) analysis showed that the recombinant proteins were predominantly trimeric in the native buffer (pH 8.0) (Fig. 2c). These data suggested that the TRAIL that coated the surface of A4 was in a functionally active form. We further validated that zTN was successfully linked to the viral capsid of A4 using a dot blot (Fig. 2d). We determined the expression levels of full-length TRAIL (z-TRIAL) and sTRAIL (z-sTRAIL) in zA4, both of which were expressed at a concentration of ~16.43 × 10^−3^ ng/mL, which was nearly twice that found in A4 (Fig. 2e). In addition, zA4 increased apoptosis by 12.24% compared with A3 (expressing TRAIL without pIX modification) and by 4.13% compared with A4 in the TRAIL-sensitive breast cancer cell line ZR-75-30 after 8 h of infection (Supplementary Fig. S1). These results indicate that zA4 carries more biologically active TRAIL than A4 after coating with sTRAIL.

### Infection by the oncolytic adenovirus vectors of AML cell lines

**Fig. 3:**
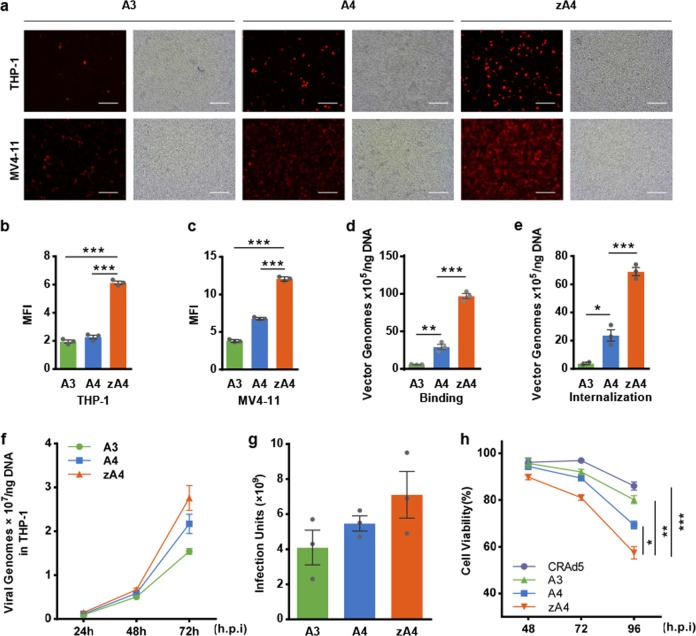
Functional validation of the recombinant Ad vectors. **a** THP-1 and MV4-11 cells were incubated with the adenovirus vectors A3, A4, and zA4 at an MOI of 100 for 16 h. The expression of RFP was detected by fluorescence microscopy. The scale bar represents 0.1 mm. **b**, **c** Quantification of infectious virus in cells in **a**. The MFI was calculated from the absolute MFI measured by Image-Pro Plus 6.0 software. **d** THP-1 cells were incubated with 1000 virus particles per cell at 4 °C for 1 h. The viral and total genomic DNA were extracted, and the adenovirus was quantified by qPCR to assess the binding ability of recombinant Ad in THP-1 cells. **e** THP-1 cells were incubated with 1000 virus particles per cell at 4 °C for 1 h and then cultured at 37 °C for 16 h. The viral and total genomic DNA were extracted, and the adenovirus was quantified by qPCR to assess the internalization of recombinant Ad in THP-1 cells. **f** THP-1 cells were infected with A3, A4, and zA4 at an MOI of 10. Infectivity was measured by quantitative real-time PCR at different time points. **g** Progeny infectious particles of A3, A4, and zA4 were collected from THP-1 cells, which were consequently used to infect HEK-293 cells. The titer was determined by the TCID_50_ method. **h** Cell viability as evaluated by the MTT assay in the recombinant Ad-infected THP-1 cell line. THP-1 cells were infected individually with progeny A3, A4, and zA4 viruses at an MOI of 10. MTT analysis was performed at 48, 72, and 96 h after infection. All data represent the mean ± SEM of three experiments. Three parallel experiments were conducted. Error bars represent the SEM. *P < 0.05; **P < 0.01; ***P < 0.001

The levels of transgene expression in TRAIL-coated oncolytic adenoviruses infecting AML cell lines were analyzed by fluorescence microscopy, as A3, A4, and zA4 had been engineered to express the RFP reporter gene. THP-1 and MV4-11 cells were infected with the three oncolytic adenovirus vectors (A3, A4, and zA4) at an MOI = 100 for 16 h. Analysis of RFP expression demonstrated that all three oncolytic adenoviruses exhibited enhanced gene transfer efficiency in MV4-11 cells in comparison with that in THP-1 cells (Fig. 3a). ZA4 produced ~2- and 3-fold increases in RFP expression compared with A3 and A4 in THP-1 cells, respectively (Fig. 3b); a similar change was also observed in MV4-11 cells (Fig. 3c). Meanwhile, all three types of oncolytic adenoviruses hardly infected H9 cells (Supplementary Fig. S2), indicating that our constructed Ad5 vectors specifically infected cancer cells but not the corresponding normal cells.

To test the infection efficiency of TRAIL-coated oncolytic adenovirus in AML cells, we carried out a cell-binding assay and assessed the internalization of the three vectors (A3, A4, zA4) in THP-1 cells. To do so, THP-1 cells were incubated with A3, A4, and zA4 at an MOI = 50 for an hour at 4 °C and the cell-bound adenoviruses were quantified by qPCR. The results showed that Ad5 binding to THP-1 cells was significantly increased when TRAIL was linked to the surfaces of recombinant adenovirus particles (Fig. 3d). Then, the cellular internalization mediated by these vectors was assessed after a 2 h incubation at 37 °C and the cell surface-bound vectors were removed by washing with 0.2 mol/L glycine (pH 2.2). We found that TRAIL significantly enhanced the cellular entry of pIX-modified vectors (A4 and zA4) into THP-1 cells, indicating that the presence of TRAIL on the viral particle surface promotes Ad5 endocytosis (Fig. 3e). Taken together, these data suggest that modification of pIX with TRAIL increases infection by the oncolytic Ad5 virus of AML cell lines.

To evaluate the replication capacity of zA4 at a lower MOI, THP-1 cells were infected with A3, A4, and zA4 at an MOI of 10. The replication efficiency was evaluated by quantitative real-time PCR after a 24, 48, and 72 h infection. (Fig. 3f). All three Ad5 viruses showed time-dependent genomic replication in tumor cells and zA4 had a higher replication capacity than A3 and A4 (Fig. 3f). We also investigated the infectivity of the progeny virus in HEK-293 cells. zA4 progeny virus showed more efficient infectivity after quantitative analysis of the virus titer (Fig. 3g). Although there was no statistical significance, the difference was not trivial, considering that the units of the *y* axis were 10^7^ and 10^9^. In line with this tendency, zA4 led to an ~10% greater reduction in cell viability than A4 at 96 h, whereas A4 reduced cell viability by 10% compared with A3 (Fig. 3h). Therefore, there was a definite trend, as zA4 showed enhanced infectivity and activity in cancer cells. As we only tested these parameters at 72 h or 96 h, it is likely that a better result could be expected after a longer incubation time. These data indicate that the progeny viruses retained their infectious and oncolytic abilities.

### Cytotoxicity of TRAIL-coated oncolytic adenovirus in AML cells in vitro

**Fig. 4:**
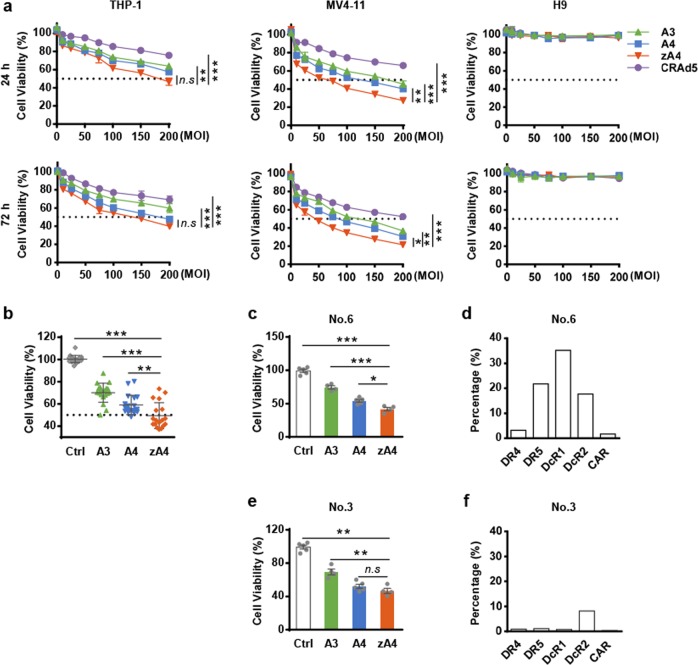
In vitro evaluation of the anti-AML activity of recombinant Ad vectors. **a** Normal cells (H9) and AML cells (THP-1 and MV4-11) were infected with recombinant Ad at different MOIs (0, 10, 25, 50, 75, 100, 150, and 200) for 24 or 72 h, followed by an MTT assay for the assessment of cell viability. All data represent the mean ± SEM of three experiments. **b** Primary AML cells were infected with recombinant Ad at an MOI of 100 for 72 h, followed by an MTT assay to assess cell viability. **c**, **e** MTT assay for the cell viability assessment of No. 6 and No. 3 cells infected by recombinant Ad. **d**, **f** The expression of relevant receptors in No. 6 and No. 3 cells. The expression of all receptors was assessed by flow cytometry. Three parallel experiments were conducted. Error bars represent the SEM. *P < 0.05; **P < 0.01; ***P < 0.001

To evaluate the anti-AML activity of the zA4 oncolytic adenovirus, THP-1 and MV4-11 cells were infected at different MOIs. The cytotoxicity was assessed by an MTT assay after 24 and 72 h of infection (Fig. 4a). We found that all three oncolytic adenovirus vectors induced dose (MOI)-dependent AML killing activity that was significantly greater than that induced by viruses that did not express TRAIL (CRAd5). However, no cytotoxicity was observed in H9 cells. Importantly, zA4 induced significant apoptosis in both the THP-1 and MV4-11 cell lines compared with A3 and A4.

To further investigate the therapeutic efficacy of the oncolytic adenovirus vectors, we analyzed the cytotoxicity of the vectors in primary AML cells from 19 clinical AML patients. The results show that all three oncolytic adenovirus vectors successfully infected and elicited cytotoxicity in primary AML cells (Fig. 4b). Overall, compared with A3 or A4, zA4 significantly suppressed the in vitro proliferation of the primary AML cells, which was particularly exemplified by the No. 6 cells (Fig. 4c), which had high expression levels of DR4, DR5, and decoy receptors (Fig. 4d). However, in some cases, such as the infection of No. 3 cells, there were no significant differences between A4 and zA4 infection (Fig. 4e), as No. 3 cells do not express death receptors (Fig. 4f). These findings indicate that modification of pIX with TRAIL and the combination of modified pIX with a replication-competent adenovirus vector results in a stronger capacity to inhibit AML cell growth.

### Tumor targeting and anti-AML activity of zA4 oncolytic adenovirus vectors in a THP-1 xenograft model

**Fig. 5:**
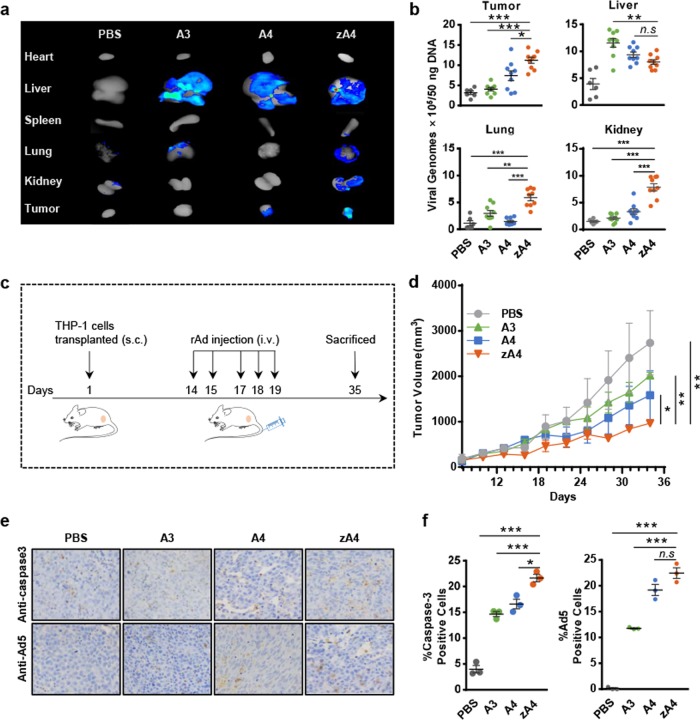
Targeting ability and antitumor effects of recombinant Ad vectors in the subcutaneous transplantation tumor-bearing model. **a** Biphotonic imaging of RFP in different tissues of tumor-bearing mice. Athymic nude mice were subcutaneously injected with THP-1 cells in the right flank. After the tumor volume reached ~100 mm^3^, the mice were i.v. injected with 5 × 10^10^ IU of A3, A4, or zA4. The mice were killed at 72 h after injection of the recombinant Ad and the RFP expression levels in tumor tissues and organs were analyzed by bioluminescent imaging. **b** The recombinant Ad in the tumor, liver, lung, and kidney was quantified by quantitative PCR. **c** Treatment scheme of the xenograft tumor model. **d** Tumor growth curve of the subcutaneous tumor-bearing mouse model. Tumor volumes were measured every 3 days and the calculated volumes (mean ± SEM) are presented in the growth curves. **e** Detection of caspase-3 activation and recombinant Ad infection in tumor tissues. Mice infected with recombinant Ad were killed at day 35 following tumor transplantation. The activation of caspase-3 and infection with recombinant Ad were detected by immunohistochemistry. **f** Statistical analysis of immunohistochemistry results. The percentages of cells positive for active caspase-3 fragments and Ad5 infection were determined in three microscopic fields in three different mice at ×200 magnification (mean ± SEM). Error bars represent the SEM. *P < 0.05; **P < 0.01; ***P < 0.001

To evaluate whether TRAIL-coated oncolytic adenoviruses can target AML cells in vivo, BALB/c nude mice were transplanted with subcutaneous THP-1 xenografts. Oncolytic adenovirus vectors were IV injected after the tumor volume reached 100 mm^3^. At 72 h after administration, the tissue distribution of the adenovirus vectors was detected by bioluminescent imaging (Fig. 5a) and the vector biodistribution in organs was analyzed with viral genome DNA analysis (Fig. 5b). Our data showed that both A4 and zA4 targeted tumor tissues and attenuated liver tropism and intrahepatic transduction. Furthermore, zA4 showed enhanced targeting ability compared with A4.

To analyze the anti-AML activity of the TRAIL-coated oncolytic adenoviruses in vivo, subcutaneous tumor-bearing BALB/c nude mouse models were treated with three different types of oncolytic adenovirus vectors (Fig. 5c). Compared with the PBS-treated mice, all three virus-treated groups demonstrated tumor growth delay (Fig. 5d). In particular, the tumor volume of zA4-treated mice was significantly smaller than that of the other treatment groups. Histological examination by H&E staining of liver and kidney tissues after oncolytic adenovirus vector treatment showed no pathological changes (Supplementary Fig. S4a). The concentrations of alanine aminotransferase (ALT) and aspartate aminotransferase (AST) in serum showed that there was no significant difference among the mice treated with A3, A4, and zA4 (Supplementary Fig. S4b). Thus, our oncolytic Ad5 viruses produced no significant liver damage. We also performed IHC analyses of caspase-3 activation and the replication of the adenovirus vectors in tumor tissues after 3 weeks of treatment (Fig. 5e). We found that zA4-treated mice showed the highest level of caspase-3 activation among all four groups, which was consistent with the finding of the highest levels of zA4 in tumor tissues. These results showed that TRAIL-coated zA4 oncolytic adenovirus could effectively replicate and express the therapeutic gene in AML cells in vivo.

**Fig. 6:**
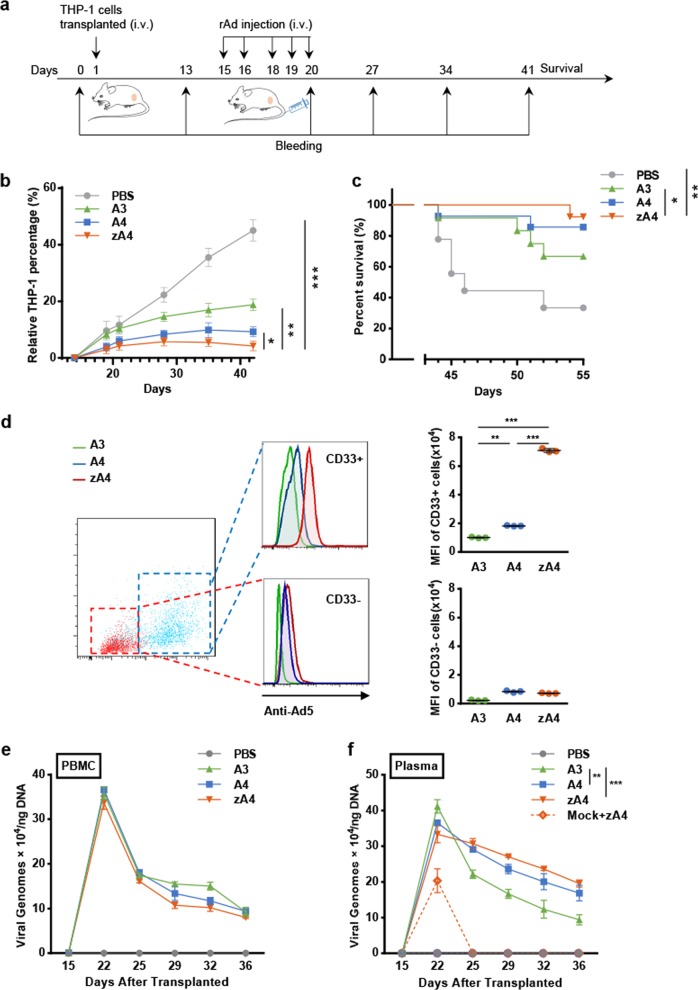
Targeting ability and anti-cancer effects of recombinant Ad vectors in the subcutaneous model. **a** Treatment strategy used in the subcutaneous model. Freshly cultured THP-1 cells (1 × 10^7^) were injected subcutaneously into the right flank of mice. Recombinant Ad (2 × 10^8^ IU) was administered intravenously each time. Each mouse was administered 1 × 10^9^ IU of recombinant Ad during the entire treatment. The mice were killed 35 days after tumor cell transplantation. **b** Tumor burden curve as indicated by the relative percentage of THP-1 cells. The proportion of THP-1 cells in the PBMCs of each mouse was detected using an anti-CD33 antibody by flow cytometry. **c** Kaplan–Meier analysis of animal end point survival after intravenous treatment with A3, A4, and zA4. **d** The targeting ability of recombinant Ad in PBMCs of tumor-bearing mice was detected by flow cytometry. The PBMCs from mice treated with A3, A4, and zA4 were gated according to staining with CD45 and CD33 antibodies. The targeting ability of the recombinant Ad was represented by RFP expression by adenoviruses. **e** Detection of viral load in PBMCs. The viral genome was detected by quantitative real-time PCR. **f** Evaluation of the viral genome in plasma. The quantity of the viral load was detected by quantitative real-time PCR. “Mock+zA4” indicates the viral load of nontumor-bearing mice with intravenous injection of zA4. Error bars represent the SEM. *P < 0.05; **P < 0.01; ***P < 0.001

In addition, we transplanted THP-1 cells via i.v. injection and established a transplantable leukemia BALB/c nude mouse model. The transplanted nude mice were monitored by flow cytometry to detect the percentage of THP-1 cells in PBMCs. The THP-1 percentage reached 15–20% on the 14th day after transplantation (Supplementary Table S3), which is when treatment was started (Fig. 6a). After the i.v. administration of the viral vectors, the percentage of THP-1 cells in PBMCs was monitored by flow cytometry every 7 days. On day 42, the number of THP-1 cells was markedly reduced by all three oncolytic adenovirus vectors compared with that in the control mice (Fig. 6b). Similar to the subcutaneous model, zA4 showed a better ability to suppress leukemia progression than A3 or A4 (Fig. 6b). ZA4 or A4 treatment significantly prolonged mouse survival (Fig. 6c). In addition, 24 h after the first virus injection, RFP expression was detected in both CD45^+^CD33^+^ THP-1 cells and mouse PBMCs. A marked shift in fluorescence was demonstrated in CD45^+^CD33^+^ cells (THP-1) from zA4-treated mice compared with that in cells from A3- and A4-treated mice (Fig. 6d), whereas a difference was not found in the CD45^+^CD33^−^ cells in mouse PBMCs. Collectively, these findings indicate that the targeted infection of THP-1 cells by TRAIL-coated oncolytic adenoviruses impairs leukemia progression and prolongs host survival. Changes in the viral load in blood were detected by qPCR. At 22 days after THP-1 transplantation, the viral load in the treated mice reached a peak and then slowly decreased at different rates (Fig. 6e). At 36 days after transplantation, the A3 load in the bloodstream reached a concentration of ~9.41/50 ng DNA; the A4 load was ~16.86/50 ng DNA and the zA4 load was ~19.55/50 ng DNA. The modest difference in the viral load in THP-1 cells might be due to the drastic dilution caused by the large number of mouse PBMCs. When we tested the viral load in plasma, zA4 was maintained at the highest levels, which may explain why zA4 showed a better antitumor ability (Fig. 6f). In addition, we also detected the viral loads of nontumor-bearing mice that were subjected to zA4 i.v. injection. When there are no tumor cells in the blood, rAd cannot exist in the plasma for a long time.

### Rh2 enhances TRAIL-induced apoptosis of AML cells

**Fig. 7:**
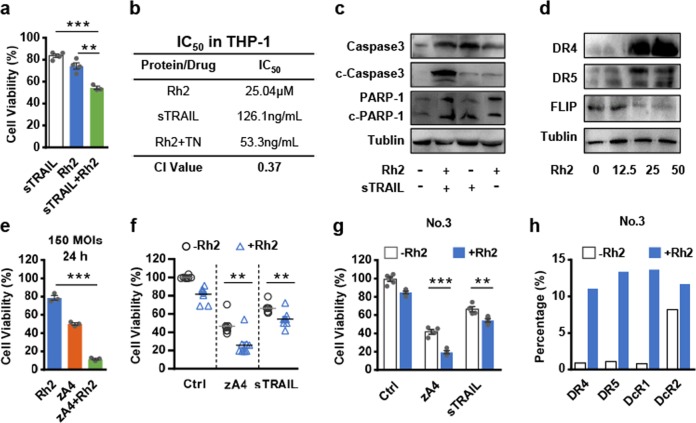
Rh2 synergizes with recombinant Ad by enhancing the expression of DR4 and DR5, and by inhibiting the expression of FLIP. **a** THP-1 cells were treated with 0.1 μM sTRAIL for 16 h, 25 μM Rh2 for 4 h, or 25 μM Rh2 for 4 h, followed by 16 h of treatment with 0.1 μM sTRAIL. Cell viability was analyzed using the MTT assay. **b** IC_50_ and CI values resulting from the combined effects of Rh2 and sTRAIL in the THP-1 cell line. **c** THP-1 cells were incubated with/without 25 μM Rh2 for 4 h and then incubated with medium with/without 0.1 μM sTRAIL for 16 h. The expression of PARP and caspase-3 was detected by western blotting with the indicated antibodies. **d** THP-1 cells were treated with different concentrations of Rh2 for 6 h. Western blot analysis was performed with anti-DR4, anti-DR5, and anti-FLIP antibodies. **e** THP-1 cells were infected with the three recombinant Ads at an MOI of 150 for 24 h. Cell viability was analyzed using the MTT assay. **f**, **g** AML primary cells were treated with 25 μM Rh2 for 4 h and then infected with recombinant Ad at an MOI of 50 for 72 h. The results for all specimens (**f**) and specimen No. 3 (**g**) are presented. **h** AML cells were incubated with/without 25 μM Rh2 for 4 h and protein expression levels were detected using flow cytometry. Three parallel experiments were conducted. Error bars represent the SEM. *P < 0.05; **P < 0.01; ***P < 0.001

Some primary AML cells do not express or express low levels of TRAIL receptors (Supplementary Table S2), which negatively influences the targeting and antitumor activity of zA4. Previous studies have shown that Rh2 can induce the expression of death receptors and cytotoxicity in leukemia cells. Therefore, we attempted to increase TRAIL-induced cell death with Rh2. Indeed, when AML cells were pretreated with Rh2 for 4 h followed by treatment with low concentrations (25 ng/mL) of sTRAIL for 16 h, cell death was significantly increased compared with that of cells treated with Rh2 or sTRAIL alone, as shown by the MTT assay (Fig. 7a). To determine whether the combined effect is synergistic, we calculated the IC_50_ value for each treatment and the combination index (CI) of the IC_50_. The CI of THP-1 cells was 0.37 (CI < 1, CI = 1, and CI > 1 indicate synergism, additivity, and antagonism, respectively), indicating that the combination of Rh2 and sTRAIL is synergistic (Fig. 7b). We also showed that combining Rh2 with TRAIL increased the activation of the caspase pathway (Fig. 7c). Then, we verified that the expression of death receptors was increased, while that of the apoptosis inhibitor FLIP was decreased by Rh2 in a dose-dependent manner (Fig. 7d).

Subsequently, THP-1 cells were infected with zA4 at an MOI = 150 for 24 h or an MOI = 50 for 72 h, to evaluate whether Rh2 can enhance the activity of TRAIL. Indeed, Rh2 significantly enhanced the killing activity of zA4 under both infection conditions (Fig. 7e and Supplementary Fig. S5). Rh2 further significantly inhibited the proliferation of primary AML cells treated with sTRAIL or zA4 (Fig. 7f, g), which induced the expression of the TRAIL-related receptors DR4, DR5, and DcR1 (Fig. 7h). Moreover, we noted that the combination of Rh2 with low doses of A3, A4, and CRAd5, but not the replicative adenovirus, was able to enhance the activity of TRAIL following viral replication (Supplementary Fig. S6).

### In vivo anti-AML effects of combined treatment with Rh2 and TRAIL-coated oncolytic adenovirus

**Fig. 8:**
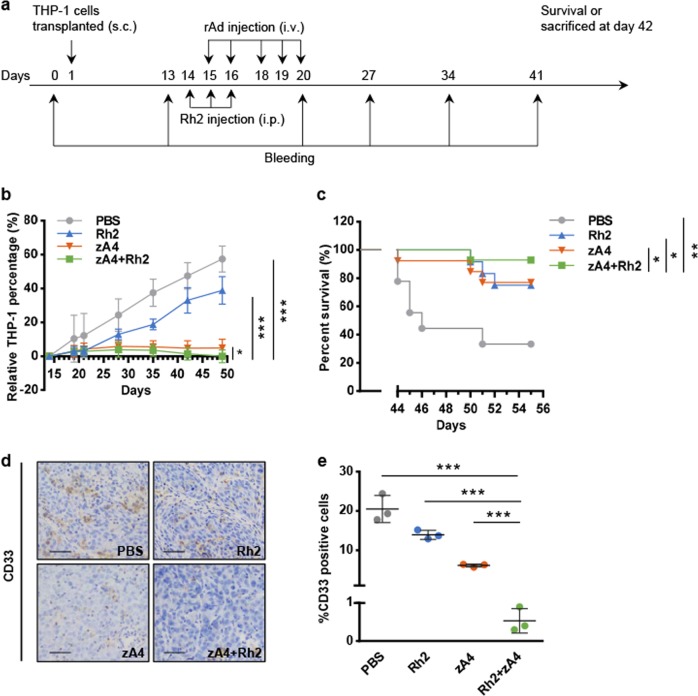
Antitumor effect of combination therapy in an intravenous tumor-bearing model. **a** Treatment strategy used for combination therapy in an intravenous tumor-bearing model. THP-1 cells (1 × 10^7^) were administered to each mouse intravenously. Recombinant Ad (2 × 10^8^ IU) was administered intravenously each time. Each mouse was administered with 1 × 10^9^ IU recombinant Ad during the entire treatment. Rh2 was administered at 5 mg/kg. Blood samples were drawn on the day before THP-1 cell transplantation and on the 13th, 20th, 27th, 34th, and 41st days after transplantation. All mice were killed 42 days after tumor cell transplantation. **b** THP-1 cell percentage in the PBMCs of each mouse was detected with an anti-CD33 antibody using flow cytometry. **c** Kaplan–Meier analysis of animal end point survival after treatment with intravenous zA4, intraperitoneal Rh2, and the combination. **d** Metastatic THP-1 cells in the liver were detected by immunohistochemistry (IHC) with an anti-CD33 antibody. The scale bar represents 0.1 mm. **e** The percentages of CD33-positive cells in three microscopic fields were quantified at ×200 magnification (mean ± SEM). Error bars represent the SEM. *P < 0.05; **P < 0.01; ***P < 0.001

BALB/c nude mice with established transplantable AML were treated with Rh2 or zA4 using a suboptimal dose of each agent (Fig. 8a). Treatment with zA4 alone stabilized the disease, whereas Rh2 alone modestly reduced the percentage of AML cells (Fig. 8b). In contrast, the combination of Rh2 and zA4 led to a reduction of the disease burden by significantly reducing AML cell counts in peripheral blood compared with Rh2 or zA4 treatment alone. Compared with the PBS treatment in control mice, coadministration of Rh2 and zA4 significantly prolonged mouse survival. Treatment with Rh2 or zA4 alone was inferior to treatment with Rh2 + zA4 in a statistically significant manner (Fig. 8c). IHC analysis showed that THP-1 infiltration in the liver was inhibited by zA4 or combination treatment, whereas treatment with Rh2 alone barely inhibited the liver infiltration of THP-1 cells (Fig. 8d, e).

## Discussion

The antitumor effect of oncolytic viruses is dependent on their ability to selectively kill tumor cells and to induce systemic antitumor immunity. Human Ad5 is a prototype oncolytic adenovirus that has been used in a number of preclinical studies and clinical trials. However, oncolytic adenoviruses have not been fully evaluated for the treatment of hematopoietic malignancies due to the absence of CAR and integrin expression on their surfaces. Qian et al.^14^ reported that although some cells from acute lymphoblastic leukemia and chronic myelogenous leukemia patients expressed various levels of CAR, all 18 samples from AML patients showed no CAR expression.^25^ Here we found that most primary leukemia cells obtained from AML patients expressed moderate levels of TRAIL-related receptors but did not express adenovirus receptors. Consequently, the presence of both death and decoy receptors enhanced the TRAIL-coated oncolytic adenovirus infection of AML cells, as zA4 demonstrated better infection and transduction efficacy than A3 and A4 in MV4-11 and some primary AML cells. This indicates that our TRAIL-coated oncolytic adenovirus vectors might potentially show good antitumor activity in most AML patients, even though some express low levels of death receptors. Furthermore, we also found that zA4 was significantly better at infecting THP-1 cells than the fiber chimeric adenovirus vectors Ad5/k3 and Ad5/k35 (Supplementary Fig. S3); fiber modifications are often used to increase the infectivity of Ad5 in many types of cancer cells with low CAR expression.^13^ Nevertheless, other types of fiber modifications (e.g., RGD insertion) can further improve the infection ability of zA4 in AML cells.

Systemic administration of viruses are needed for AML treatment with oncolytic virotherapy. However, effective i.v. administration of Ad5-based vectors is hampered by the complex interactions between virus and host proteins, leading to hepatic tropism and immune clearance, and causing the Ad5 vector to be diverted away from the desired tumor targets.^25–28^ As death receptors are expressed at higher levels on the surfaces of tumor cells than on normal cells, TRAIL has been used to target tumor cells. Pan et al.^28^ showed that TRAIL can be conjugated with a toxic small molecule for targeted delivery of the desired chemical to tumor cells. Another study also used TRAIL to enhance the targeted antitumor activity of nanocarriers by conjugating TRAIL onto their surfaces.^26^ Our biodistribution analysis showed that zA4 showed improved tumor targeting and reduced liver enrichment in a THP-1 subcutaneous tumor-bearing model (Fig. 4a, b). In addition, we also found that TRAIL-coated oncolytic adenovirus vectors selectively infect THP-1 cells in a transplantable AML BALB/c nude mouse model (Fig. 5d), which also suggests the potential for i.v. therapy of AML. Considering the differences between humans and mice, and how various adenovirus–host interactions in the blood involving complement proteins,^29^ coagulation factors,^30^ and natural antibodies^31^ may affect viral tropism, further studies are warranted.

Through the analysis of venous and subcutaneous tumor-bearing models, treatment with zA4 was shown to have a significant antitumor effect. Although most primary AML cells expressed TRAIL receptors and a previous study showed that adenoviruses expressing full-length TRAIL could overcome TRAIL resistance in several cancer cell lines,^32^ relatively low levels of TRAIL receptor expression in some AML cells limited apoptosis induction by TRAIL-coated oncolytic adenovirus vectors. Chemotherapy drugs may sensitize cells to TRAIL-induced apoptosis through enhancing a cross-talk between internal and external pathways involved in cell death. TRAIL-based combination therapy is a promising strategy to treat cancer.^33^ Therefore, TRAIL-coated adenovirus might potentially overcome resistance and improve efficacy when combined with chemotherapy. The ginsenoside Rh2 can enhance the response of tumor cells to TRAIL to a certain extent. The active components of ginseng mainly include ginsenoside, ginseng polysaccharide, and ginseng polypeptide. Among these components, ginsenosides have been proven to have a variety of biological activities, such as antitumor activity and improved effects on immunity.^34^ Molecular mechanism studies have shown that Rh2 promotes transcriptional activation of p53 and the activation of caspase 8 by upregulating downstream pro-apoptotic genes (*FAS*, *DR4*, and *DR5*) to induce the apoptosis of HeLa cells and human leukemia cells. In HL-60 cells, Rh2 induces apoptosis via caspase 8- and caspase 9-dependent pathways by upregulating tumor necrosis factor expression.^35^ In this study, we found that Rh2 improved the tumor-killing effect of TRAIL by activating the caspase pathway. Mechanistically, Rh2 increases the expression of TRAIL receptors, especially death receptors, in primary AML cells. Rh2 also enhances virus infection. The combination therapy (zA4 + Rh2) led to a clear regression of tumor burden, whereas zA4 alone only stabilized the disease (Fig. 8b). However, the disease progressed when treated with Rh2 alone (Fig. 8b). These data suggest that the combination therapy showed better efficacy than monotherapy, which has significant clinical relevance. Minimal residual disease is often the cause of cancer relapse. Therefore, complete eradication of AML cells with combination therapy is critical to patient survival. Our data indicate that application of TRAIL-coated oncolytic adenovirus vectors in combination with Rh2 may be an option for the treatment of relapsed AML or patients who are ineligible for intensive therapy.

Although the antitumor mechanism underlying the effects of oncolytic viruses is not fully understood, it is clear that oncolytic viruses can cause lysis of tumor cells after infection. In solid tumors, the release of tumor-derived antigens due to tumor lysis could stimulate and promote an antitumor immune response. Due to the lack of a replication-permissive animal model for the evaluation of oncolytic Ad vectors,^36^ the in vivo activity of the oncolytic adenovirus in this study was only assessed in immunodeficient mice, in which antitumor immunity cannot be evaluated. Chimeric antigen receptor T cells and checkpoint inhibitors might represent promising immunotherapeutics for AML.^37^ Oncolytic virus immunotherapy has also become a potential strategy for treating AML.^38–40^ Shen et al.^41^ reported that combining an oncolytic VSV encoding IFNβ with PD-L1 blockade enhanced the therapeutic outcomes of the treatment of murine AML. In addition to inducing tumor cell apoptosis, TRAIL also plays a role in immune regulation, although the impact of the TRAIL/TRAIL-R system on the immune environment of cancer is unclear.^42,43^ Therefore, defining the effect of TRAIL expressed on oncolytic viruses on antitumor immunity is critical for further application of TRAIL-coated oncolytic adenovirus vectors, and we will evaluate their antileukemia activity in permissive immunocompetent animal models in future studies.

In summary, the TRAIL-coated oncolytic adenovirus vector zA4 is active against primary AML cells from patient peripheral blood or bone marrow and shows potential for use in the targeted treatment of AML by i.v. administration. Moreover, combination therapy with Rh2 and zA4 enhances the therapeutic effects in both AML cells and a transplantable leukemia BALB/c nude mouse model. This study provides a rationale for further development of oncolytic adenoviruses as a new therapeutic strategy to treat hematopoietic malignancies such as AML.

## Supplementary information

Supplementary Information

## Data Availability

The datasets analyzed during the current study are available from the corresponding author upon reasonable request.
