# Supplementary material for: Enhancing the antitumor activity of an engineered TRAIL-coated oncolytic adenovirus for treating acute myeloid leukemia
Source: Signal Transduct Target Ther. 2020 Apr 24;5:40. doi: 10.1038/s41392-020-0135-9 (PMC7181830; doi:10.1038/s41392-020-0135-9)
Supplement: Supplementary file 1 — Supplementary Information [file 41392_2020_135_MOESM1_ESM.docx]

Supplementary Materials for

**Enhanced targeting anti-tumor activity of a TRAIL-coated oncolytic adenovirus for treatment of acute myeloid leukemia**

**Zixuan Wang ^a^, Wenmo Liu ^a^, Lizheng Wang ^a^, Peng Gao ^c^, Zhe Li ^a^, Jiaxin Wu ^a^, Haihong Zhang ^a^, Hui Wu ^a^, Wei Kong ^a, b^, Bin Yu ^a, *^, Xianghui Yu ^a, b, *^**

^a^ National Engineering Laboratory for AIDS Vaccine, School of Life Sciences, Jilin University, Changchun 130012, China

^b^ Key Laboratory for Molecular Enzymology and Engineering, the Ministry of Education, School of Life Sciences, Jilin University, Changchun 130012, China

^c^ Department of Hematology, Jilin Province People’s Hospital, Changchun 130021, China

*contributed equally

Corresponding authors.

Address: School of Life Sciences, Jilin University, Changchun 130012, China.

Fax: +86 0431 85167751.

E-mail addresses: yubin@jlu.edu.cn (B. Yu), xianghui@jlu.edu.cn (X. Yu)

**This document include:**

Table S1 to S3, and Figure S1 to S7.

**Table S1.** Receptor expression in healthy donors. DR4, DR5, DcR1, DcR2, CAR, Interginαvβ3, and Interginαvβ5 were detected with specific antibodies using flow cytometry. “-“ indicates ‘not detected’.

**Table S2.** Receptor expression in AML cells. DR4, DR5, DcR1, DcR2, CAR, Interginαvβ3, and Interginαvβ5 were detected with specific antibodies using flow cytometry.

| Days After Planted | THP-1(%) |
| --- | --- |
| 0 | 0 |
| 7 | 1.2±0.1 |
| 14 | 17.15±2.15 |
| 21 | 22.13±6.03 |
| 28 | 38.95±8.35 |
| 35 | 53.58±12.81 |
| 42 | 64.42±6.74 |
| 49 | 72.61±7.42 |
| 56 | 75.12±10.02 |

**Table S3.** Tumor growth curve of venous tumor-burdened model. The proportion of THP-1 cells in the PBMCs of each mouse was detected using the anti-CD33 antibody by flow cytometry.

**2 h**

**4 h**

**8 h**

**A3**

**A4**

**zA4**


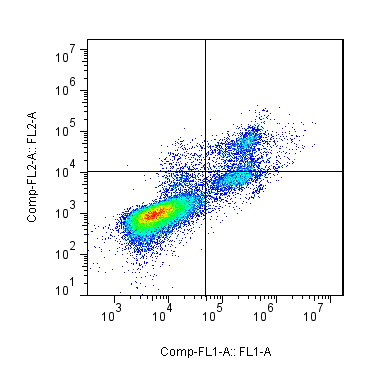

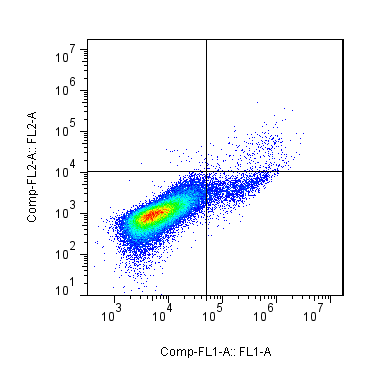

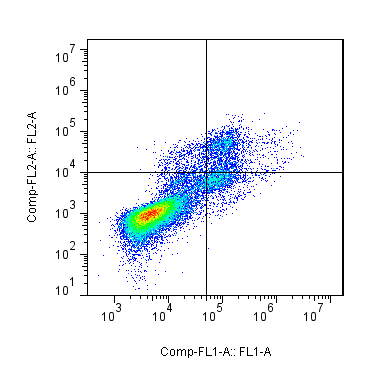

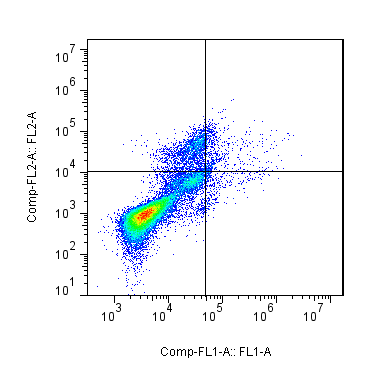

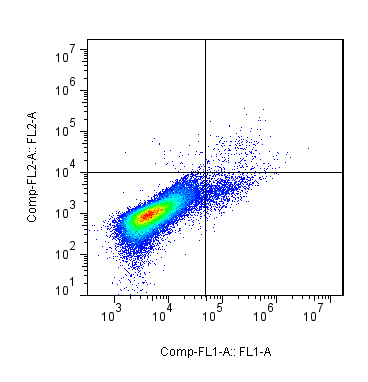

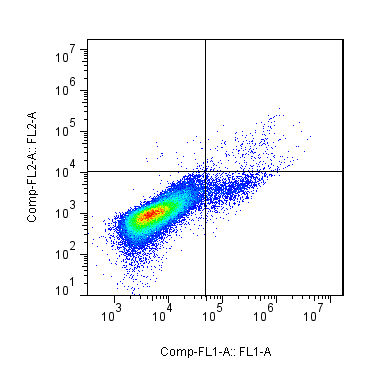

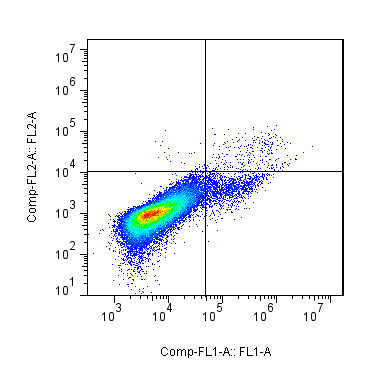

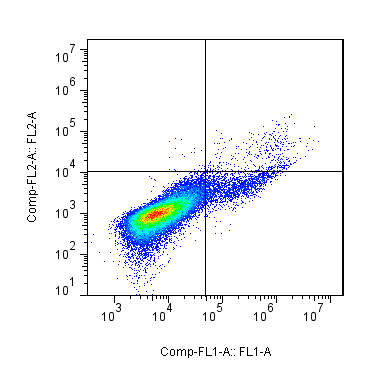

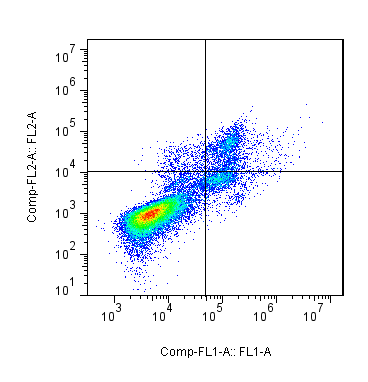


**1.92%**

**1.77%**

**0.69%**

**3.07%**

**0.73%**

**3.48%**

**0.58%**

**2.60%**

**0.95%**

**3.98%**

**6.61%**

**5.97%**

**0.79%**

**3.70%**

**7.48%**

**5.64%**

**8.83%**

**7.62%**

**Figure S1.** Anti-tumor ability of the oncolytic adenoviruses on ZR-75-30. ZR-75-30 cells treated with three viruses (A3, A4, zA4) at 100 MOI for 2, 6 and 8 hours. The cells were analyzed by the apoptosis assay kit and flow cytometry.


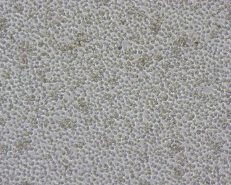

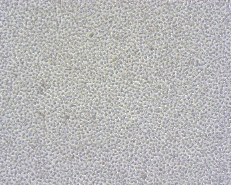

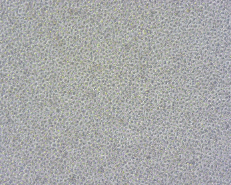

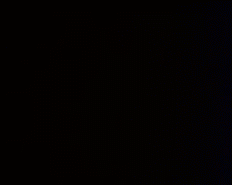

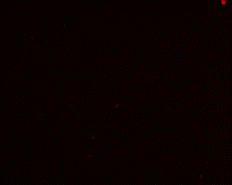

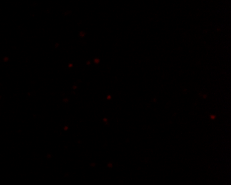


A3

A4

zA4

**Figure S2.** Infection differences among three oncolytic adenoviruses in H9. H9 cells were infected with A3, A4, or zA4 at an MOI of 100 for 16 hours. Expression of RFP was detected by fluorescence microcopy.

**Figure S3.** Differences in the infection ability of for oncolytic adenoviruses in THP-1. Ad5/k3 and Ad5/k35 were generated based on Ad5 in our laboratory according to Sirena. D et al^1^. The chimeric virus was generated by replacing the knob protein with the knob protein of Ad3 or Ad35, respectively. Ad5/k3 carried only the Ad3 knob at the extremity of a serotype 5 fiber bound to serotype 5 penton base, as well as Ad5/k35.THP-1 was infected by the rAds in 200 MOIs for 16 hours. The infection ability was measures by quantity real-time PCR. Three parallel experiments were conducted for all experiments. Error bars represent SEM. * P < 0.05; **P< 0.01; ***P< 0.001.


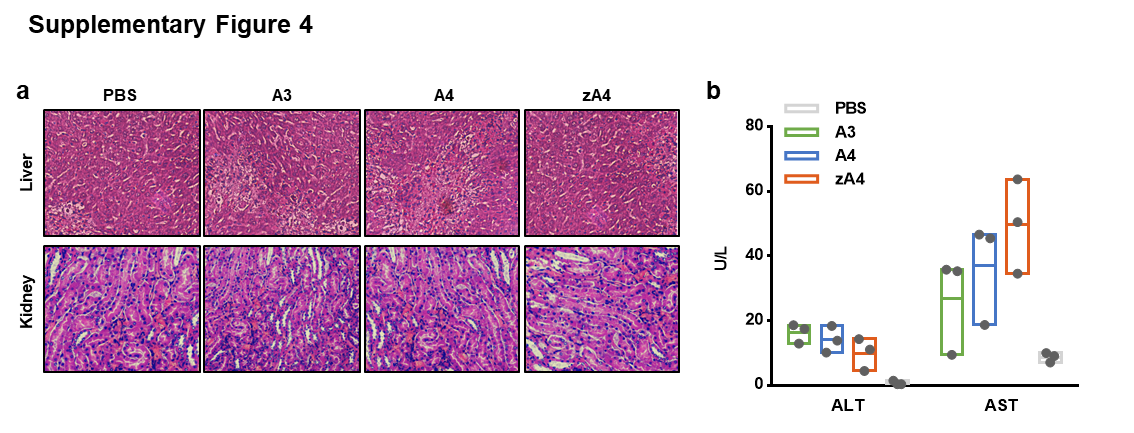


**Figure S4.** Detection of liver and kidney damage of tumor-bearing mice. (a) Liver metastatic nodules were examined by paraffin embedding, sectioning, and H&E staining. (b) ALT and AST concentration in serum was detected at 42 days after THP-1 transplanted


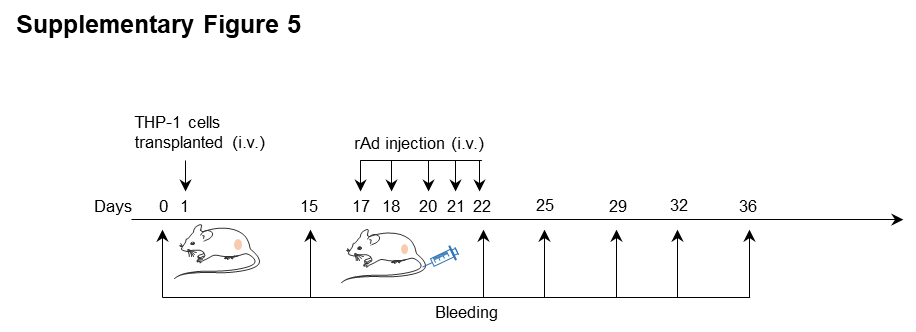
 **Figure S5.** Treatment strategy of intravenous transplantation tumor-bearing model. 1 × 10^7^ THP-1 cells were administered in each mouse subcutaneously. 2 × 10^8^ IU recombinant Ads were administered intravenously each time. Each mouse was administered 1 × 10^9^ IU recombinant Ads during the entire treatment. Blood samples were taken at 0, 15, 22, 25, 29, 32, 36 days after tumor transplanted. Each treatment group contain 3 mice.

**Figure S6.** Recombinant Ads can synergize with Rh2 in the THP-1 cell line. Cell viability was analyzed using the MTT assay. THP-1 cells were infected with three recombinant Ads at an MOI of 50 for 72 hours.

**Figure S7.** Viability of A3, A4, and CRAd5 infected THP-1 cells. THP-1 cells were incubated with/without 25 μm Rh2 for 4 hours, and then infected with A3, A4, or CRAd5 at an MOI of 50 for 72 hours. Cell viability was analyzed using the MTT assay.

**Reference**

1 Sirena, D. *et al.* The nucleotide sequence and a first generation gene transfer vector of species B human adenovirus serotype 3. *Virology*. **343**, 283-298, (2005).
